# Special Issue “Molecular Biomarkers in Cancers: Advances and Challenges”

**DOI:** 10.3390/ijms27188242

**Published:** 2026-09-16

**Authors:** Paramjit S. Tappia, Bram Ramjiawan

**Affiliations:** 1 Asper Clinical Research Institute, St. Boniface Hospital, Winnipeg, MB R2H 2A6, Canada; bramjiawan@sbrc.ca; 2 Department of Food and Human Nutritional Sciences, Faculty of Agricultural and Food Science, University of Manitoba, Winnipeg, MB R3T 2N2, Canada; 3 Department of Pharmacology and Therapeutics, Max Rady College of Medicine, Rady Faculty of Health Sciences, University of Manitoba, Winnipeg, MB R3E 0T6, Canada

Cancer remains one of the most formidable challenges in contemporary medicine, due to not only its substantial and growing global burden but also the profound biological heterogeneity that characterizes individual tumors [1,2,3]. Although advances in imaging, histopathology, molecular biology, and targeted therapeutics have substantially improved cancer management, an urgent need remains for reliable biomarkers capable of facilitating early detection, improving prognostic assessment, identifying biologically aggressive disease, and guiding therapeutic decision-making [2,3,4,5,6]. The ten articles considered in this collection provide a timely perspective on this rapidly evolving field. Collectively, they illustrate how the integration of circulating biomarkers, proteomics, transcriptomics, extracellular vesicles, microRNAs, genetic variation, and signaling pathways is progressively reshaping our understanding of cancer biology and advancing the field toward more individualized approaches to patient care [4,5,6,7,8,9,10,11]. A major theme emerging from these studies is the ongoing search for biomarkers that can complement, and potentially overcome limitations associated with, conventional diagnostic approaches. This is particularly relevant for cancers in which current screening or prognostic tools lack sufficient specificity. In prostate cancer, for example, the limited cancer specificity of prostate-specific antigen (PSA) remains an important clinical concern because elevated PSA can lead to unnecessary biopsies and the detection of clinically indolent disease. Ferre-Giraldo et al. (contribution 1) explored small extracellular vesicles (sEVs) present in semen as a source of molecular information. Their study demonstrated the differential expression of selected tRNA-derived small RNAs (tRFs) in men with prostate cancer and suggested that combining selected tRFs with PSA could improve discrimination between malignant and indolent disease, particularly for clinically significant tumors.

A complementary investigation by the same group (contribution 2) examined transcriptomic signatures in semen sEVs. Differential expression of KLK3 and PCA3, together with associations involving CREB3L4, CCNQ, and DUSP23, provided evidence that semen-derived extracellular vesicles may contain molecular information relevant to tumor aggressiveness. Importantly, classifiers based on combined transcript levels demonstrated potential value when used independently or in conjunction with PSA and semen citric acid measurements. These findings are noteworthy because they exemplify an important movement in oncology: the transition from simply detecting the presence of cancer toward determining its biological and clinical significance. A biomarker that can distinguish aggressive disease from indolent disease has considerably greater clinical value than one that merely indicates the possibility of malignancy.

Endometrial cancer provides another important example. Pušić et al. (contribution 3) investigated circulating AKR1B1 and AKR1B10 as potential prognostic biomarkers. Although their study was preliminary and involved a relatively modest cohort, higher plasma AKR1B10 levels were observed in patients with Grade 3 disease, while patients with lower levels of both AKR1B1 and AKR1B10 demonstrated more favorable overall and recurrence-free survival. The authors appropriately characterize these observations as hypothesis-generating and emphasize the need for prospective validation. Similarly, Monasta et al. (contribution 4) demonstrated the potential of tissue washings as a biological source for biomarker discovery in endometrial cancer. Using data-independent acquisition mass spectrometry-based proteomics, the investigators identified numerous proteins capable of distinguishing controls from cancer patients and high-grade from low-grade disease. Of particular interest were PRRC2A and SYDE2, which were validated in an independent cohort and implicated in pathways associated with tumor growth. Taken together, these studies emphasize that the choice of biological specimen is becoming as important as the biomarker itself. Blood, semen extracellular vesicles, and tissue washings represent minimally invasive or readily accessible sources from which tumor-associated molecular information may be obtained. Such approaches could ultimately facilitate repeated monitoring of disease while reducing dependence on invasive tissue sampling.

A second major theme is the increasing recognition that biomarkers are most informative when they are connected to the underlying biology of tumor development and progression. The study by Skrzypek et al. (contribution 5) illustrates this principle through an investigation into the genetic variation in xenobiotic-metabolizing enzymes and endometrial cancer susceptibility. Variants in NAT2, CYP1A1, and CYP2D6 were associated with increased endometrial cancer risk, whereas a NAT1 variant demonstrated a potentially protective association. Importantly, the investigators also observed increased CYP1A1 and CYP2D6 expression in tumor tissues. These findings raise the possibility that inherited genetic variation and altered metabolism of endogenous or exogenous compounds may interact in determining carcinogenic susceptibility. The importance of signaling pathways is further demonstrated in hepatocellular carcinoma (HCC). García-Gaytán et al. (contribution 6) examined metabotropic glutamate receptor 3 (mGluR3) during progression from fibrosis and cirrhosis to HCC. Increasing mGluR3 expression was associated with disease progression and correlated with inflammatory mediators, including IL-6 and TGF-β. Increased glutamate concentrations were also observed, while functional studies suggested that mGluR3 activation can influence cellular viability. These observations position mGluR3 at an intersection between altered metabolism, inflammation, and tumor-cell survival.

The relationship between molecular alterations and tumor phenotype is also evident in gastro-entero-pancreatic neuroendocrine neoplasms. Cavalcanti et al. (contribution 7) identified four miRNAs that were downregulated in Grade 2 compared with Grade 1 tumors and demonstrated an association with the FGF/FGFR signaling pathway. The increased expression of FGFR2 in Grade 2 tumors provides biological support for the observed miRNA changes and raises the possibility that molecular signatures could complement conventional histological grading. Likewise, Kasperczak et al. (contribution 8) examined BCL9 and TPX2 in clear-cell renal cell carcinoma. Both proteins were increased in tumor tissue, with BCL9 associated with shorter progression-free survival and TPX2 associated with shorter overall survival. Their respective relationships with Wnt signaling and cell-cycle regulation further reinforce the concept that prognostic biomarkers may simultaneously provide insights into the biological processes driving tumor progression. These investigations collectively reinforce an important principle: the most valuable biomarkers may be those that function not simply as statistical indicators of disease but also as molecular reflections of the biological processes responsible for disease progression.

The third theme emerging from this collection concerns the complex interaction among tumor cells, the immune system, and the tumor microenvironment. This relationship is particularly important as immunotherapy becomes increasingly integrated into cancer treatment. Guinart-Cuadra et al. (contribution 9) investigated soluble immune checkpoints in patients with extensive-stage small-cell lung cancer receiving chemotherapy and anti-PD-L1 therapy. Several soluble immune checkpoint molecules, including sPD-L1, sPD-L2, BTLA, HVEM, TIM-3, and CD27, were elevated in patients compared with healthy donors. Higher concentrations of selected soluble checkpoints were also associated with liver metastases and poorer prognosis. The observed relationships between soluble immune checkpoints, leukocyte–platelet complexes, and PD-L1 expression suggest that circulating immune markers may provide information concerning the systemic immune state as well as tumor–immune interactions.

The study by Huang et al. (contribution 10) takes this concept further by examining hypoxia-mediated resistance to PD-L1 inhibition in HCC. Their work identifies NOXA as an important component of the response to hypoxic conditions and demonstrates that NOXA suppression is associated with reduced apoptosis in immunotherapy-resistant cells. The findings suggest that hypoxia, apoptosis regulation, and immune resistance are interconnected processes and raise the possibility that NOXA could represent a therapeutic target for improving immunotherapy responsiveness. These observations are particularly relevant because therapeutic resistance remains one of the principal barriers to successful cancer treatment. The identification of biomarkers that predict resistance before treatment, or that reveal the mechanisms responsible for resistance during treatment, could permit a more rational selection of therapeutic strategies.

An important emerging direction in cancer biomarker research is the integration of multiple biological measurements with advanced computational approaches, including machine learning. Such approaches recognize that the biological complexity and heterogeneity of cancer may not be adequately captured by a single biomarker. Rather, combinations of metabolic, molecular, clinical, and demographic features may provide a more comprehensive representation of disease risk and enable the identification of subtle signatures associated with early-stage malignancy. Our own investigations in lung and breast cancer have similarly explored the potential of biomarker-based approaches in conjunction with machine learning methodologies for improving early cancer detection and risk stratification [12,13]. These studies support the broader concept that the future of cancer biomarker research may lie not only in identifying individual molecules associated with disease but also in determining how multidimensional biomarker profiles can be integrated to generate clinically meaningful predictive models. Importantly, such approaches must ultimately be subjected to rigorous validation in appropriately powered and independent cohorts before their incorporation into routine clinical practice.

While the findings presented across these studies are encouraging, an important distinction must be maintained between biomarker discovery and clinical validation. Several of the investigations are explicitly preliminary, involve relatively modest sample sizes, or require independent validation. For example, the endometrial cancer study involving AKR1B1 and AKR1B10 reported limited discriminative performance for predicting lymphovascular invasion and concluded that the model was not suitable for clinical application in its current form. This caution is essential. Modern omics technologies can identify an enormous number of statistically significant molecular differences between tumors and controls. However, clinical biomarkers must satisfy substantially more demanding criteria. They must demonstrate reproducibility across populations, analytical robustness, adequate sensitivity and specificity, incremental value over existing clinical parameters, and, most importantly, clinical utility. A biomarker that performs well in a discovery cohort but does not improve clinical decision-making has limited translational value.

An additional challenge is the movement from single biomarkers toward integrated signatures. Several of the studies in this collection demonstrate that combinations of molecular markers can provide greater discriminatory or prognostic value than individual markers. This is evident in the prostate cancer studies involving PSA and semen-derived molecular signatures, as well as in the endometrial cancer investigation combining AKR1B1, AKR1B10, and clinical variables. The future of precision oncology is therefore unlikely to depend exclusively on individual biomarkers. Rather, for many clinical applications, multidimensional molecular signatures integrated with clinical, pathological, and demographic information may provide more informative approaches to diagnosis, prognosis, and therapeutic stratification.

The ten articles assembled here provide a representative snapshot of the rapidly expanding landscape of cancer biomarker research. Despite considerable diversity in tumor type and experimental approach, a common message emerges: cancer is a biologically complex disease that cannot always be adequately characterized by conventional histopathology or a single circulating marker. The studies highlight several promising directions, from circulating AKR1B proteins and soluble immune checkpoints to proteomic signatures, extracellular vesicle-associated transcripts and tRFs, miRNA-FGFR interactions, xenobiotic-metabolizing enzymes, mGluR3, NOXA, BCL9, and TPX2. Together, they demonstrate how advances in molecular profiling are providing increasingly detailed insight into tumor biology while simultaneously creating opportunities for improved diagnosis, prognostication, and therapeutic stratification.

In conclusion, the enthusiasm for emerging biomarkers must be balanced by scientific rigor. Discovery represents only the first step. Prospective validation in sufficiently large and diverse populations, standardized analytical methodologies, assessment against established clinical markers, and the demonstration of meaningful clinical benefit will ultimately determine whether these molecular signatures progress from promising research findings to clinically useful tools. The central challenge for the coming years is therefore not simply to discover more cancer biomarkers but to identify which biomarkers provide actionable information that changes patient management and improves outcomes. The studies presented in this collection contribute important pieces to that larger objective and underscore the increasingly central role of molecular biology in the evolution of precision oncology.

We extend our sincere appreciation to all contributors, each of whom is highly regarded in their respective fields, for their valuable contributions to this Special Issue. Collectively, these contributions provide a timely overview of the evolving landscape of cancer biomarker research and highlight the diverse approaches being employed to improve cancer detection, prognostic assessment, and therapeutic stratification. We trust that this Special Issue will be of interest to investigators and clinicians actively engaged in cancer research, as well as to readers seeking a broader understanding of the emerging role of biomarkers in oncology. Importantly, we hope that the studies presented herein will stimulate further research into the identification, characterization, and validation of biomarkers specific to individual cancers and their clinically relevant subtypes, with particular emphasis on the early detection of disease. Continued efforts in this direction will be essential for translating promising molecular and cellular signatures into reliable and clinically meaningful tools that can contribute to more precise and individualized cancer management. We also anticipate that this Special Issue will serve as a useful resource for medical students, clinical fellows, graduate students, and other trainees seeking to broaden their knowledge and understanding of the biological basis, clinical relevance, and future applications of cancer biomarkers.

## Data Availability

No new data were created or analyzed in this study. Data sharing is not applicable to this article.
